# Antiepileptic Drug Management in Acute Ischemic Stroke: Are Vascular Neurologists Utilizing Electroencephalograms? An Observational Cohort Study

**DOI:** 10.1155/2020/6250531

**Published:** 2020-12-19

**Authors:** Rahul Rao, Dominique J. Monlezun, Tara Kimbrough, Brian J. Burkett, Alyana Samai, Sheryl Martin-Schild

**Affiliations:** Stroke Program, Department of Neurology, Tulane University School of Medicine, New Orleans, LA, USA

## Abstract

**Introduction:**

This study examines the utility of electroencephalography (EEG) in clinical decision making in acute ischemic stroke (AIS) patients in regards to the prescription of antiseizure medications.

**Methods:**

Patients were grouped as having positive EEG (+) for epileptiform activity or negative EEG (-). These studies were no more than 30 minutes in length. Patients' charts were retrospectively reviewed for antiepileptic drug (AED) use before, during, and on discharge from AIS hospitalization.

**Results:**

Of the 509 patients meeting inclusion criteria, 24 (4.7%) had a positive EEG. Patients did not significantly differ with respect to any demographic or baseline characteristics with the exception of prior history of seizure. In the EEG- group, AEDs were discontinued in only 3.5% of patients. In the EEG+ group, only 37.5% of patients had an initiation or change to their AED regimen within 36 hours of the study. 62.5% of the EEG+ group had a cortical stroke. *Significance*. Our results indicate that vascular neurologists are not using spot EEGs to routinely guide inpatient AED management. EEGs may have greater utility in those with a prior history of seizures and cortical strokes. Longer or continuous EEG monitoring may have better utility in the AIS population if there is clinical suspicion of seizure.

## 1. Introduction

Seizures are well-known complications of acute ischemic stroke (AIS) [1–3]. About 10% of all stroke patients experience seizures from stroke onset to several years later [1]. It is also the most commonly identified cause of epilepsy in adults greater than 35 years old [4]. Electroencephalographic (EEG) changes associated with cerebral vessel thrombosis have been reported as early as the 1940's [5]. Studies have suggested that epileptiform activity on EEG increases the risk of poststroke seizure, and this has been associated with an unfavorable outcome [6, 7]. However, the employment of EEG in guiding antiepileptic drug (AED) management in stroke patients is unclear. In one study of intracerebral hemorrhage, less than 20% of EEGs were ordered to monitor clinically suspected seizure activity [8]. The indication and utility of EEGs ordered of AIS patients is largely unknown. One study from 2019 showed that EEG performed in the ED may help identify patients with large acute strokes and may correlate with infarct volume [9], but with modern imaging techniques and interruption of stroke workflow, the utility of an EEG in ischemic stroke needs to be brought into question.

Our study sought to identify whether EEG results in AIS patients are being used to guide clinical decision making in regards to the prescription of AEDs and benzodiazepines. As of 2017, no systemic review on AED use in epilepsy related to ischemic stroke had been published [10]. We hypothesize that inpatient vascular neurologists are not using EEG results to guide AED regimens.

## 2. Methods

### 2.1. Demographics

The study design was a retrospective cohort study using a prospectively collected single-center database in New Orleans, Louisiana, United States of America. Inclusion criteria were age 18 years or older, admitted to the Tulane Medical Center stroke service, AIS diagnosis, and one or more EEG evaluations during the same stay admission. These studies were between 20 and 30 minutes in length. Subjects were excluded from this study if there was no EEG performed during their admission for acute stroke.

### 2.2. Variables

The independent variable was EEG ever positive (EEG+), which is defined as whether a patient ever had an EEG that had any epileptiform discharges or seizure activity on the official read. The primary outcome was inpatient antiseizure medication use. Secondary outcome measures were antiseizure use before and after EEG results and benzodiazepine use. This study did not require any variables from follow-up appointments.

### 2.3. AED Classification

The medications used as part of secondary outcome variables were classified as follows.

Carbamazepine, divalproex sodium (Depakote)^∗^, valproic acid (Depakene), ethosuximide, fosphenytoin, lacosamide, lamotrigine, levetiracetam (Keppra), oxcarbazepine, phenytoin (Dilantin), phenobarbital, topiramate^#^, and vigabatrin were all considered AEDs. Chlordiazepoxide (Librium), clorazepate, clonazepam (Klonopin), diazepam (Valium), and flurazepam were all considered long-acting benzodiazepines. Alprazolam (Xanax), estazolam, lorazepam (Ativan), midazolam (Versed), oxazepam, and temazepam were all considered short-acting benzodiazepines.

^∗^Unless specifically noted in the medical record as treatment for bipolar disorder.

^#^Only at doses > 200 mg daily.

### 2.4. Statistical Analysis

Descriptive statistics were performed for the entire sample. Bivariable analysis based on positive EEG was conducted with independent sample *t*-test comparing means and Wilcoxon rank-sum tests comparing medians for continuous variables as appropriate, and Pearson's chi-square test or Fisher's exact test comparing proportions for categorical variables as appropriate. Multivariable logistic regression was used to assess the possible independent association between positive versus negative EEG and the study outcome measures. Predictive margins were calculated for the fully-adjusted, final regression model to investigate significant predictors. The final regression models were reviewed by an academic physician and academic biostatistician/data scientist to ensure support by substantive clinical and statistical theory and evidence. Correlation matrix and variance inflation factor were utilized to ensure no multicollinearity in the final models. All regression estimates with 95% confidence intervals (CIs) are reported as fully adjusted results. Statistical significance was set at a two-tailed *p* value < 0.05. All analyses were conducted using STATA 14.2 (STATACorp, College Station, Texas, USA). Approval for this study was obtained through the Institutional Review Board of Tulane University (study number 533461).

### 2.5. Standard Protocol Approvals, Registrations, and Patient Consents

Approval from an ethical standards committee to conduct this study was received.

## 3. Results

At our medical center, 509 patients out of 1807 patients (28.1%) who were admitted between 09/2008 and 01/2015 had at least one EEG performed after AIS. Of these, 24 (4.7%) had at least one EEG that was positive (EEG+) and 485 (95.3%) never had epileptiform discharges, seizure tendency, or overt seizures on their EEG read (EEG-). Patients did not significantly differ with respect to any demographic or baseline characteristics with the exception of prior history of seizure of which 37.5% of EEG+ patients had a positive history of seizure which was seen in only 8.0% of EEG- patients (*p* < 0.001). AEDs were discontinued in only 17 (3.5%) of EEG-patients by discharge. While 3.71% of the EEG- had an initiation of or a change to their AED regimen within 36 hours of the EEG read, this frequency only rose to 37.5% of EEG+ patients (*p* < 0.001). 67% of EEG- patients and 79.2% of EEG+ patients were administered short-acting benzodiazepines during their inpatient stays (*p* = 0.214). Of the 24 patients with a positive EEG, 15 (62.5%) of them had a cortical stroke (Table 1).

The multivariate regression of medication use by positive EEG examined the covariates of age, gender, race, and a history of seizures. EEG+ patients had greater odds (OR: 5.84, 95% CI: 2.08-16.43, *p* < 0.001) of receiving inpatient AEDs compared to those with EEG- (Table 2). EEG+ had a positive predictive correlation with inpatient AED use.

A past history of seizures led to greater odds (OR: 20.34, 95% CI: 8.29-49.95, *p* < 0.001) of receiving AEDs compared to those without such a history. Inpatient AED administration did not differ among patients of different ages, race, or gender, but home AED regimen was significantly correlated to increasing age (*p* = 0.037). EEG+ patients have greater odds of having AEDs started or changed both before (OR: 4.03, 95% CI: 1.49-10.92, *p* = 0.006) and after (OR: 11, 95% CI: 3.90-30.99, *p* < 0.001) the EEG results were read. There was no significant difference between EEG+ and EEG- patients having their AEDs discontinued within 36 hours after the EEG read. EEG+ patients had greater odds (OR: 8.11, 95% CI: 2.65-24.79, *p* < 0.001) of having AEDs as part of the discharge regimen compared to EEG- patients. EEG+ patients had greater odds (OR: 2.82, 95% CI: 1.02-7.80, *p* < 0.045) of receiving long-term inpatient benzodiazepines compared to EEG- patients.

## 4. Discussion

At our medical center and countless around the country, there are no standardized guidelines as to when to order EEG or AEDs. In the ischemic stroke population, this presents a major problem as these patients have a lowered seizure threshold. Conversely, many of these patients may have AEDs prescribed due to clinical suspicion of seizure, but whether EEGs are being used to help guide these decisions is unclear.

In our cohort, patients with a past history of seizures or epilepsy had greater odds of being on home AEDs and getting inpatient AEDs. Similarly, patients with a history of seizures had greater odds of receiving inpatient AEDs before the EEG read returned, most likely due to the reasonable decision to maintain home medications.

More than one in four AIS patients screened with EEG due to suspected seizures were started on an AED before the EEG result returned, indicating that many physicians are likely treating seizures mainly based on clinical findings. EEG+ patients had greater odds of having AEDs started or changed before the EEG results were read indicating that clinical judgement on seizure tendency is likely sound. Furthermore, our results indicate that EEGs are largely not being used to change AED management in the AIS population.

Only 3.5% of the EEG- population had their AED discontinued after a negative EEG was reported. Between the EEG+ and EEG- groups, the odds of discontinuation did not differ significantly. This suggests that physicians were so convinced that a patient had a seizure that a negative EEG did not dissuade them. Therefore, an EEG is not providing significant value in patients for whom seizure is highly suspected and clinical suspicion may be a more sensitive test in determining AED use. In fact, some scales designed to predict poststroke seizures do not include EEG [11]. Alternatively, inpatient vascular neurologists at this center may have felt uncomfortable discontinuing AED regimens once they had begun. This suggests that AEDs may be prescribed unnecessarily in many patients and EEGs are not providing a line of defense to discontinue these medications.

Only 37.5% of EEG+ patients had an initiation of AED medication or a change in dosage to their existing medications after the EEG read. This further illustrates that medication decisions are not being influenced by EEG, even if there is seizure activity or tendency. This begs the question, why are we ordering inpatient spot EEGs when they are not going to help guide management?

Benzodiazepine use was very frequent in this population, with 79.2% of EEG+ patients and 67% of EEG- patients receiving at least one during their in-hospital stays. Benzodiazepines are historically used to treat status epilepticus but they are also very commonly used now as a sedative and not necessarily to thwart epileptiform activity [12–14]. It is not always clearly documented whether benzodiazepines are used for antiseizure activity or sedation, and sometimes, physicians may try and cover both with this type of drug. Future studies should explore for what indications benzodiazepines were used following a stroke.

Studies have shown that lesion site and size is directly related to the risk of seizure occurrence [15, 16]. All of the positive EEG patients in this study had a cortical stroke. The underlying mechanism explaining this association may be related to excitatory and inhibitory imbalance in the cerebral cortex caused by damage to the cortex [17]. We did not compare these findings to our total sample so we are unable to determine if EEG+ patients are more likely to have a cortical stroke compared to all patients who presented to our medical center with an ischemic stroke. Further studies are needed to determine the frequency of seizures in those with subcortical ischemic strokes.

The multivariate regression analysis clearly showed that EEG+ had greater odds of receiving inpatient AEDs than EEG-, an expected trend. A patient with a past history of seizures had greater odds of being on home AEDs and getting inpatient AEDs. Similarly, patients with a history of seizures had greater odds of receiving inpatient AEDs before the EEG read returned, most likely due to the reasonable decision to maintain home medications. Ordering an inpatient EEG in an AIS patient with a prior history of stroke may have a higher yield compared to those without a seizure history.

However, while the amount of patients discharged with an AED was significantly higher in the EEG+ population, it was still only 66.7%. Our study did not examine the duration that these patients stayed on AEDs. It is possible that many of them continued on these medications indefinitely, which may be unnecessary or they were discontinued at outpatient follow-up.

There is a paucity of data in examining EEG utility in the AIS population. While the effectiveness of specific AEDs and their adverse effects in this group has been reported; currently, no studies have examined the prevalence of overall AED prescription in this population [18]. Shorter duration or spot EEGs are not consistently used to initiate, change, or discontinue AEDs in our cohort. In this era of minimizing unnecessary testing, we should start by limiting a test that does not clearly guide medication management in the acute ischemic stroke population.

## 5. Limitations

This was a single-institution study. A multicenter, prospective study may be needed to completely answer some of these questions. After all, no controlled trials evaluating only poststroke seizures have been conducted to evaluate specific agents.

The EEGs that were used were limited in duration, not exceeding 30 minutes in length. It was also not entirely clear in our chart review if EEGs were done during an ictal or interictal phase for these patients nor is clear why there were ordered in the first place. It is possible that there was clinical suspicion of seizure that made the provider order an EEG, but other reasons could have included prolonged monitoring, poor neurological exam, or provider preference.

Longer or continuous EEGs (cEEG) may be of higher yield and clinical utility in monitoring stroke patients. One study demonstrated that findings from cEEG led to changes in AED prescribing in over half of the studies performed [19]. Longer or continuous EEGs (cEEG) may be of higher yield and clinical utility in monitoring stroke patients. We did not include a baseline Glascow Coma Scale or mental status exam in our demographics section, which would have been helpful to determine if there was suspicion for nonconvulsive status epilepticus and need for more prolonged EEG monitoring.

A significant limitation of this study is that we did not specifically examine clinical suspicion of seizure and AED use. As such, we cannot confirm that an EEG was ordered when there was clinical suspicion of seizure. We also cannot determine if patients who received an AED after EEG+ did so exclusively because of the EEG result.

We do not have data on whether patients received an AED due to clinical suspicion of seizure prior to EEG. This is important as AEDs before an EEG result may suppress epileptiform discharges [20], which may falsely categorize certain patients into the nonseizure category (EEG-). We also did not evaluate statin prescription in our group, which may be important as a recent study has suggested lowering of poststroke epilepsy risk with statin use [21].

Gabapentin was not considered an AED in this study, as it is frequently used to treat neuropathic pain [22]. Benzodiazepines were also not included as an AED but were instead their own category as they too are used for other reasons. In certain cases, they are used as AEDs. We also did not evaluate medical complications in these patients or ascertain patient satisfaction. These factors may also lead to changes in AED management that should be factored into future studies. Finally, we did not correlate our outcomes with stroke severity and etiology. As mentioned before, the size of the stroke may influence the development of poststroke seizures [15, 16]. Ischemic stroke etiology such as infective endocarditis may suggest an increased risk for seizures, which may necessitate more extensive electrographic monitoring of these patients.

## 6. Future Directions

Poststroke seizures are an unfortunate complication of an already devastating neurological injury. Comparing patients who received an EEG and those who did not in the AIS population may further help determine if there are any predictive factors that allow EEGs to be of higher yield in this vulnerable population.

While many of these patients may benefit from AEDs, future studies should aim to identify risk factors or circumstances that favor seizure recurrence and development of epilepsy. These patients may benefit from close hospital follow-up, prolonged EEG monitoring, and AED trials. Examination of whether these patients remain on AEDs after hospital follow-up would also shed light on whether AEDs are being unnecessarily prescribed. Hopefully, more evidence on EEG and AED use in this population can ultimately lead to guidelines on when to order them.

## Figures and Tables

**Table 1:** Descriptive statistics of the overall sample by unique subject and bivariable analysis by EEG result (*N* = 509)^∗^.

Covariate	Sample	EEG ever positive		*p* value
	*N* = 509	No: *n*1 = 485 (95.28%)	Yes: *n*2 = 24 (4.72%)	
Demographics				
Age, mean (SD)	65.18 (14.87)	65.15 (14.85)	65.79 (15.55)	0.837
Female, no. (%)	241 (47.72)	230 (47.82)	11 (45.83)	0.849
African American, no. (%)	350 (69.03)	331 (68.53)	19 (79.17)	0.271
Past medical history, no. (%)				
Seizure	48 (9.43)	39 (8.04)	9 (37.50)	<0.001
Stroke	217 (43.31)	206 (43.19)	11 (45.83)	0.798
Diabetes	191 (39.30)	182 (39.39)	9 (37.50)	0.853
Hypertension	418 (83.77)	400 (84.03)	18 (78.26)	0.463
Carotid stenosis	14 (3.50)	14 (3.62)	0 (0.00)	1.000
Coronary artery disease	114 (23.03)	110 (23.31)	4 (17.39)	0.620
Atrial fibrillation	63 (12.70)	61 (12.90)	2 (8.70)	0.754
Congestive heart failure	70 (16.75)	67 (16.63)	3 (20.00)	0.725
Daily alcohol	30 (7.41)	28 (7.14)	2 (15.38)	0.249
Alcoholism	35 (8.64)	32 (8.16)	3 (23.08)	0.093
Substance abuse	14 (15.56)	13 (16.05)	1 (11.11)	1.000
Admission, mean (SD)				
Modified Rankin score	1.31 (2.05)	1.28 (2.01)	2.14 (3.01)	0.121
Temperature	36.69 (0.59)	36.69 (0.59)	36.73 (0.77)	0.811
NIHSS	10.93 (9.23)	10.84 (9.20)	12.91 (9.73)	0.292
Glucose, median (range)	122 (101-166)	122 (101-168)	113 (102-148)	0.401
Magnesium	1.83 (0.33)	1.83 (0.33)	1.79 (0.38)	0.582
White blood cells, median (range)	8.2 (6.5-10.6)	8.1 (6.5-10.6)	9.3 (7.1-12.5)	0.177
EEG, mean (SD)	0.06 (0.35)	0 (0.0)	1.3 (1.0)	<0.001
Medications, no. (%)				
Antiseizure	148 (29.08)	130 (26.80)	18 (75.00)	<0.001
Home	43 (8.72)	37 (7.84)	6 (28.57)	0.001
Ordered				
Before EEG result	136 (26.72)	120 (24.74)	16 (66.67)	<0.001
Discontinued	24 (4.72)	23 (4.74)	1 (4.17)	1.000
After EEG result (<36 hr)				
Meds started/changed	27 (5.30)	18 (3.71)	9 (37.50)	<0.001
Meds discontinued	19 (3.73)	17 (3.51)	2 (8.33)	0.224
Discharged with meds	77 (16.92)	63 (14.52)	14 (66.67)	<0.001
36 hr changes	3 (0.59)	2 (0.41)	1 (4.17)	0.135
Benzodiazepine				
Short-acting	344 (67.58)	325 (67.01)	19 (79.17)	0.214
Long-acting	58 (11.39)	52 (10.72)	6 (25.00)	0.032
Stroke, no. (%)				
Cortical	15 (62.50)	0 (0.00)	15 (62.50)	<0.05

^∗^Abbreviations: EEG: electroencephalogram. no.: number; SD: standard deviation; NIHSS: National Institutes of Health Stroke Scale; hr: hour.

**(a):** 

Covariates	Antiseizure						Given			Discontinued		
	Inpatient			Home								
	OR	95% CI	*p* value	OR	95% CI	*p* value	OR	95% CI	*p* value	OR	95% CI	*p* value
EEG positive	5.84	2.08-16.43	0.001	1.05	0.23-4.87	0.948	4.03	1.49-10.92	0.006	0.77	0.09-6.31	0.809
Age, years	1.00	0.99-1.02	0.956	0.97	0.94-1.00	0.037	1.00	0.99-1.02	0.726	0.99	0.97-1.02	0.715
Female	0.97	0.63-1.50	0.883	1.57	0.60-4.11	0.361	0.90	0.57-1.40	0.631	1.24	0.53-2.90	0.617
Black	1.44	0.89-2.33	0.140	1.24	0.43-3.56	0.692	1.62	0.98-2.70	0.061	1.24	0.48-3.24	0.654
Past seizures	20.34	8.29-49.95	0.000	119.65	43.76-327.15	0.000	19.62	8.39-45.84	0.000	1.56	0.43-5.69	0.501
	Antiseizure after EEG result						Antiseizure at discharge					
	Started/changed			Discontinued			Given			Given after 36 hr changes		
	OR	95% CI	*p* value	OR	95% CI	*p* value	OR	95% CI	*p* value	OR	95% CI	*p* value
EEG positive	11.00	3.90-30.99	0.000	2.74	0.55-13.63	0.218	8.11	2.65-24.79	0.000	10.70	0.72-158.86	0.085
Age, years	0.98	0.96-1.01	0.253	1.01	0.97-1.04	0.734	0.99	0.97-1.01	0.398	0.98	0.91-1.05	0.597
Female	0.99	0.42-2.31	0.973	0.89	0.34-2.33	0.820	0.79	0.43-1.45	0.455	0.62	0.05-7.65	0.707
Black	3.30	0.94-11.56	0.062	0.68	0.26-1.81	0.439	1.48	0.75-2.92	0.260	0.15	0.01-1.97	0.147
Past seizures	3.29	1.21-8.90	0.019	1.01	0.21-4.92	0.993	21.12	9.68-46.06	0.000	3.67	0.25-52.85	0.340

**(b):** 

	Benzodiazepine					
	Short			Long		
	OR	95% CI	*p* value	OR	95% CI	*p* value
EEG positive	2.02	0.72-5.66	0.182	2.82	1.02-7.80	0.045
Age, years	0.99	0.98-1.00	0.076	0.98	0.96-1.00	0.057
Female	0.73	0.50-1.07	0.106	0.73	0.42-1.34	0.329
Black	1.00	0.66-1.51	0.998	0.75	0.42-1.34	0.329
Past seizures	0.78	0.40-1.50	0.452	1.10	0.44-2.73	0.835

## Data Availability

Data is available from Dr. Monlezun by reasonable request.
